# Supplementary material for: High-Dimensional Cytometry Dissects Immunological Fingerprints of Idiopathic Inflammatory Myopathies
Source: Cells. 2022 Oct 21;11(20):3330. doi: 10.3390/cells11203330 (PMC9601098; doi:10.3390/cells11203330)
Supplement: Supplementary file 1 [file cells-11-03330-s001.zip › Suppl_Tables.docx]

**Supplementary Table 1: Antibodies used for flow cytometric analysis of PBMCs**

| **Supplementary Table 1: Antibodies used for flow cytometric analysis of peripheral blood mononuclear cells** | | | |
| --- | --- | --- | --- |
| **Fluorochrome** | **Antigen** | **Clone** | **Company** |
| BrilliantViolet650 | CD3 | UCHT1 | Biolegend |
| ECD | CD56 | N901 | Beckman Coulter |
| BrilliantViolet785 | CD4 | Okt4 | Biolegend |
| APC-AlexaFluor700 | CD8 | B9.11 | Beckman Coulter |
| BrilliantViolet650 | CD27 | O323 | Biolegend |
| FITC | CD45RA | ALB11 | Beckman Coulter |
| PC | CD196 | B-R35 | Beckman Coulter |
| PC5.5 | CD194 | L291H4 | Biolegend |
| FITC | CD183 | G025H7 | Biolegend |
| KO525 | CD146 | TEA 1/34 | Beckman Coulter |
| APC | CXCR5 | J252D4 | Biolegend |
| PE | PD-1 | EH12.2H7 | Biolegend |
| BrilliantViolet780 | CD161 | HP-3G10 | Biolegend |
| PE | IL-23R | HLT2736 | Biolegend |
| PC5.5 | CD31 | WM59 | Biolegend |
| PE | FoxP3 | 206D | Biolegend |
| APC-A700 | CD127 | R34.34 | Beckman Coulter |
| Violet660 | CD25 | BC96 | Biolegend |
| APC | CD226/DNAM-1 | 11A8 | Biolegend |
| PE | CD39 | REA739 | Miltenyi |
| Alexa Fluor 700 | CD14 | M5E2 | Biolegend |
| APC-AlexaFluor700 | CD19 | J3-119 | Beckman Coulter |
| FITC | CD1c | L161 | Biolegend |
| PE | CD69 | FN50 | Biolegend |
| BrilliantViolet510 | HLA-DR | L243 | Biolegend |
| FITC | IgD | IA6-2 | Biolegend |
| KO525 | IgM | MHM-88 | Biolegend |
| PC5.5 | CD38 | LS198-4-3 | Beckman Coulter |
| PB450 | CD21 | B-ly4 | BD Biosciences |
| ECD | CD24 | ALB9 | Beckman Coulter |
| BrilliantViolet780 | CD11c | 3.9 | Biolegend |
| PE | IgG4 | QA16A15 | Biolegend |
| APC | IgG1 | QA16A12 | Biolegend |
| BrilliantViolet660 | CD80 | 2D10 | Biolegend |
| FITC | CX3CR1 | 2A9-1 | Biolegend |
| FITC | CD223/LAG3 | 7H2C65 | Biolegend |
| APC | TIM3 | A18087E | Biolegend |
| APC | CD253 | RIK-2 | Biolegend |
| C=385 | CD18 | TS1/18 | Biolegend |
| FITC | Perforin | dG9 | Biolegend |
| PECy7 | Granzyme A | GzA-3G8.5 | BD Biosciences |
| PE | Granzyme B | REA226 | Miltenyi |
| FITC | Granzyme K | GM6C3 | SantaCruz |
| eFluor660 | Granzyme M | 4B2G4 | Biosciences |
| PE | CD34 | 581 | Biolegend |
| PerCP | NKp46 PC5.5-A | 9E2 | Biolegend |
| KO525 | CD117 | 95C3 | Beckman Coulter |
| BrilliantViolet780 | CD62L | DREG-56 | Biolegend |
| FITC | KIR | HP-MA4 | Biolegend |
| PE | CD57 | HCD57 | Biolegend |
| PE | NKG2C | S19005E | Biolegend |
| Biotin | CD337 / NKp3 | AF29-4D12 | Milteny |
| FITC | IFN-γ | 4S.B3 | Biolegend |
| FITC | CD223/LAG3 | 7H2C65 | Biolegend |
| FITC | CD107a | H4A3 | BD Biosciences |
| FITC | Perforin | dG9 | Biolegend |
| PE | CD57 | HCD57 | Biolegend |
| PE | MCP-1/CCL2 | 2H5 | Biolegend |
| PE | TNFα | MAb11 | Biolegend |
| PE | Granzyme B | REA226 | Miltenyi |
| ECD | CD56 | N901 | Beckman Coulter |
| ECD | CD16 | 3G8 | Beckman Coulter |
| PE-Cy5.5 | CD3 | UCHT1 | Beckman Coulter |
| PerCP-Cy5.5 | CD45RO | UCHL1 | Biolegend |
| PerCP-Cy5.5 | IL-6 | MQ2-13A5 | Biolegend |
| PerCP-Cy5.5 | KLRG1 | SA231A2 | Biolegend |
| PE-Cy7 | CX3CR1 | 2A9-1 | Biolegend |
| PE-Cy7 | CTLA-4/CD152 | BNI3 | Biolegend |
| APC | CD4 | 13B8.2 | Beckman Coulter |
| APC-Cy7 | CD8 | HIT8a | Biolegend |
| PacificBlue | CD8 | B9.11 | Beckman Coulter |
| BrilliantViolet510 | CD4 | OKT4 | Biolegend |
| BrilliantViolet510 | HLA-DR | L243 | Biolegend |
| BrilliantViolet510 | CD57 | QA17A04 | Biolegend |

**Supplementary Table 2: Flow cytometric panels used for PBMC analysis**

| **Supplementary Table 2: Flow cytometric panels used for PBMC analysis** | |
| --- | --- |
| **Panel** | **Antigens** |
| Basic panel (Figure 2) | CD3, CD4, CD8, CD19, CD20, CD56, live/dead marker |
| CD8 T cell panel 1 (Figure 3) | CD3, CD4, CD8, CD27, CD45ro, CD56, CD226, CD57, CD28, live/dead marker |
| CD8 T cell panel 2 (Figure 4A, 4B, 4C) | CD3, CD4, CD8, CD27, CD45ro, CD56, PD-1, TIM3, LAG3, CD18, live/dead marker |
| CD8 T cell panel 3 (Figure 4D, 4E) | CD3, CD4, CD8, CD27, CD45ro, GM-CSF, IL-17A, IL-22, IL-4, IFN- γ, TNF-α, live/dead marker |
| CD4 T cell panel (Figure 5) | CD3, CD4, CD8, CD27, CD45ro, GM-CSF, IL-17A, IL-22, IL-4, IFN- γ, TNF-α, live/dead marker |
| B cell panel (Figure 6) | CD19, CD20, IgM, IgD, IgG1, IgG4, CD21, CD24, CD27, CD38, CD80, live/dead marker |
| T helper cell panel (Suppl. Fig. 1) | CD3, CD4, CD8, CD146, CCR4, CCR6, CXCR3, ICOS, PD-1, live/dead marker |
