# Supplementary figures and images for: High-Dimensional Cytometry Dissects Immunological Fingerprints of Idiopathic Inflammatory Myopathies
Source: Cells. 2022 Oct 21;11(20):3330. doi: 10.3390/cells11203330 (PMC9601098; doi:10.3390/cells11203330)

A

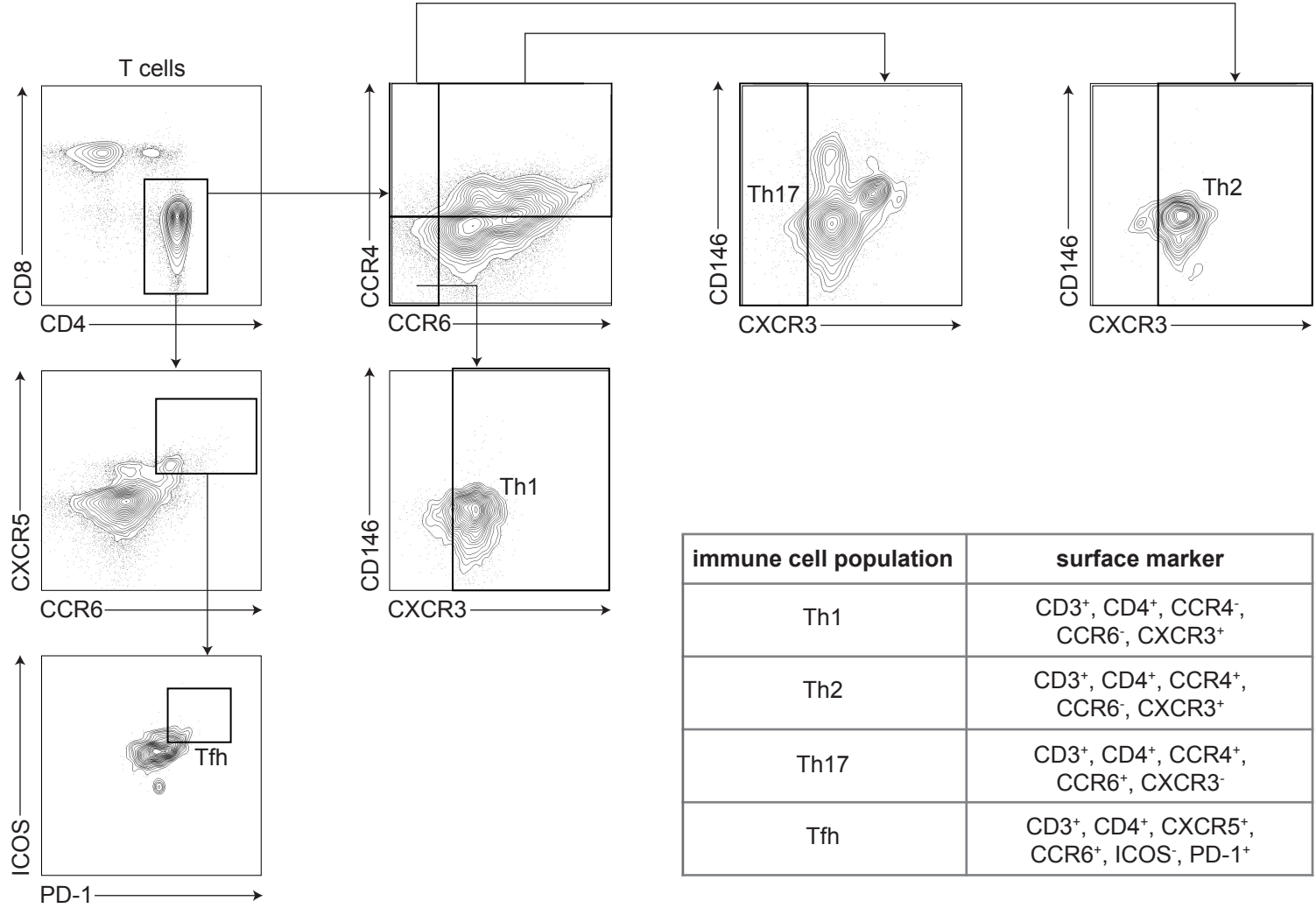

B

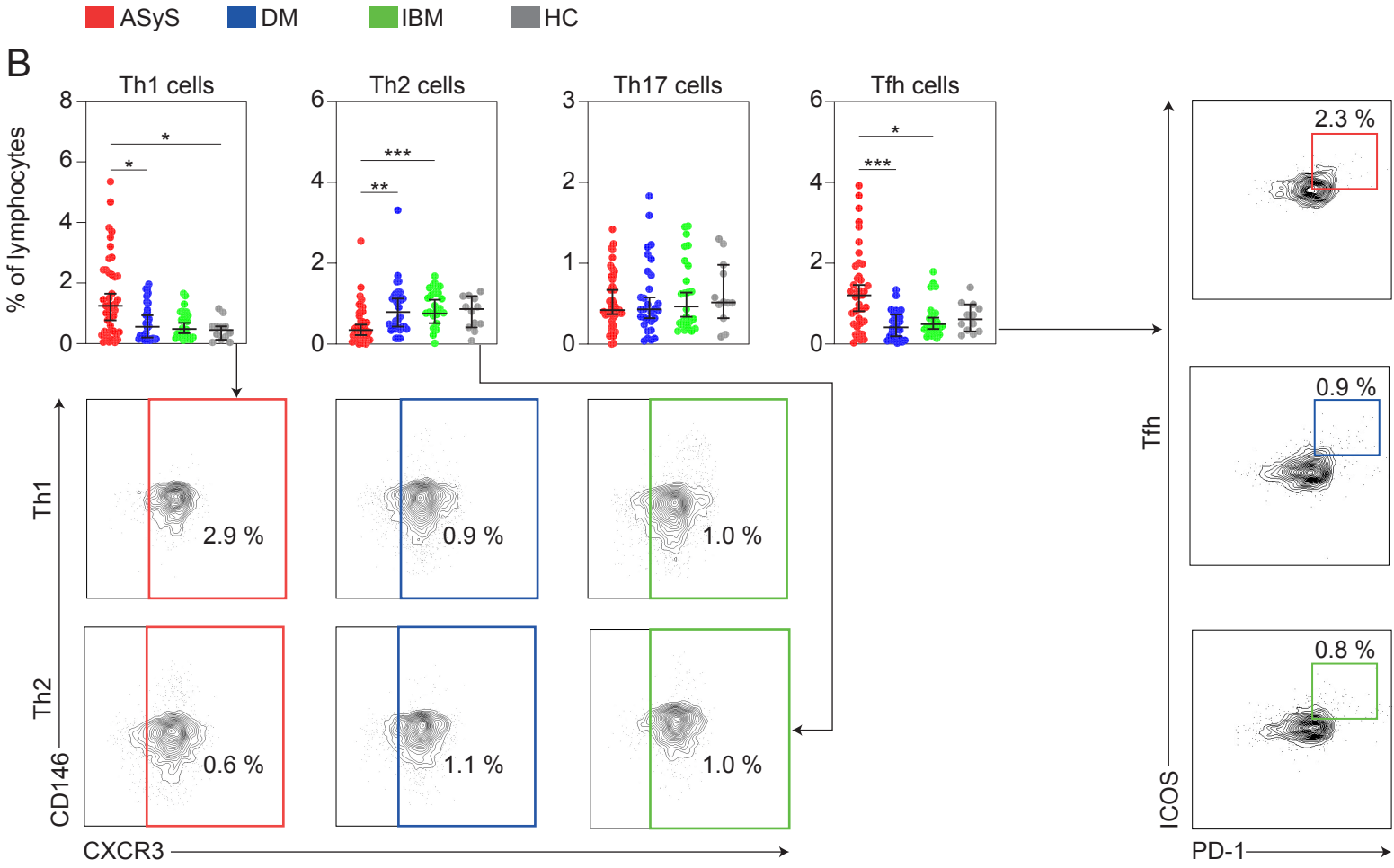

Supplement: Supplementary file 1 [file cells-11-03330-s001.zip › Suppl Fig_1.pdf]
